# The Aim of Life

**Published:** 1878-06

**Authors:** 


### THE AIM OF LIFE.

THE chief ambition of most young men of intelligence and energy, on entering the great field of the world, is to accumulate money enough to enable them to retire from business, and pass the latter years of life in quiet comfort. On a minute inquiry as to the meaning they attach to that expression, it will be found that it is to have a plenty of every thing, except that of having a plenty to do of what is necessary to be done. They want to be placed in a position which will allow them to do something, any thing, or nothing, according to the inclination of the moment. This is an aim at once narrow-minded, selfish, and dangerous; dangerous to soul, body, and estate; dangerous alike to social position, and to moral character. That very activity, energy, and enterprise which enable a man to "retire on a fortune" at fifty, and be compelled to do comparatively nothing, will as certainly make a wreck of mind and body, as that the fleetest locomotive in the world will be shivered to atoms if it is instantaneously arrested in its progress. But there is this difference between man and machinery; the magnificent engine may be gradually brought to a perfect standstill, and can be put in motion again to accomplish other labors new and grand; not so with the machinery of the mind; in its "connections" with a material body it has acquired a "momentum" in half a century's progress, a habit of action, which can not be arrested, can not be brought to a dead stand, to a position of having nothing to do, and doing nothing, without the wreck of mind or ruin of body, if indeed not both.

The only way in which a man can "retire on a fortune" with safety, with comfort, with happiness and honor, is to lay his plans so that his time shall be fully and compulsorily occupied in advancing the well-being of others, in every way compatible with the safety of his own fortune and health. It may be instructive to know the way to death which many successful business men travel, the steps taken as seen by an observant physician, the little things which lead to grand results, the total subversion of the aims and labors of a lifetime. A man retired on a fortune has nothing to do after he has built his house, laid out his grounds, and arranged his affairs perfectly to his

"own notion," according to his own "ideas of comfort." The mind can no more be arrested in its activities, than can a star in space. He gets tired of sitting about; gets tired of reading; gets tired of riding around his "place;" gets tired of visits and visitors; then the greatest pleasure, the one which can be looked forward to several times every day, is that of eating; it in time becomes, to a certain extent, the only pleasure; it is indulged in; after a while, the surplus not being worked off, the appetite either fails, or discomfort attends its indulgence, and there being nothing to do but for the mind to dwell on these discomforts, they become exaggerated, and nine times out of ten a sip of brandy is resorted to; nine times out of ten it alleviates, and having an alleviant so easily accessible, it is not at all wonderful that it should be frequently resorted to, so frequently indeed that before the man is aware of it, or even his watchful wife, he is a regular drinker, is "uncomfortable" without it; the appetite for it grows apace; he is a confirmed and hopeless drunkard.

About five years ago an enterprising firm was engaged in a lucrative business on Water street. Its integrity in business was beyond suspicion or cavil. The promptness with which its obligations were met, was the subject of general encomium, and its paper had, in every case, the value of bank-notes or of specie. The firm was composed of two members, both of them wealthy. With time their riches grew apace, and with cash their kindness and integrity increased. The senior partner resided in a magnificent west-end mansion, surrounded by all the luxuries which money could command and taste could ask. The junior partner lived with his family in a rural district upon a small farm. He passed the business hours in his establishment upon Water street, and in the cool of the evening rested in his cottage. His children grew up healthy and contented, and all the fireside virtues gamboled about his feet.

In the lapse of time the firm dissolved. Its purposes had been subserved in the success of its speculations, and the preservation of its integrity, and each partner retired to his home to enjoy the profits of his labor. The west-end millionaire has forfeited the respect and friendship of his ancient partner. We passed him last evening in a state of bloated intoxication, filthy with exposure and absolute want. The men with whom he once associated would blush to-day to recognize him. His fortune has been squandered in continued excesses, his family is scattered and penniless, and the sole aim of his degraded ambition is to find the wherewithal to purchase drink. The junior partner has not changed in circumstances. The home ties have proved stronger with him than the attractions of vice, and he still lives to demonstrate the advantage of retired virtue and contented competence.

Instead, then, of aiming to pass the latter part of life in dangerous, inglorious ease, let the ambition be to spend it in active benevolences, happifying alike the heart of both giver and receiver, thus leaving a name behind, not written in the sands of selfish indulgence, but engraven in imperishable characters on the grateful memories of man.

